# Correction: *SETD1B* mutations confer apoptosis resistance and BCL2 independence in B cell lymphoma

**DOI:** 10.1084/jem.2023114310082026c

**Published:** 2026-10-23

**Authors:** Ana Portelinha, Shenqiu Wang, Sara Parsa, Man Jiang, Alexander N. Gorelick, Sagarajit Mohanty, Soumya Sharma, Elisa de Stanchina, Marjan Berishaj, Chunying Zhao, James Heward, Neeraj K. Aryal, Omid Tavana, Jiayu Wen, Jude Fitzgibbon, Ahmet Dogan, Anas Younes, Ari M. Melnick, Hans-Guido Wendel

Vol. 221, No. 10 | https://doi.org/10.1084/jem.20231143 | September 5, 2024

The authors regret that in Fig. S3 N of their original article, the first three dot plot images (CD19, GL7, and IgD) in the third row of *VavPBcl2;SgLacZ* were mistakenly duplicated from the third row of *VavPBcl2;SgSETD1B*. The original and corrected figures are shown here. The error affects only the images; the quantification and statistics were derived from the raw data and are unchanged, and the correction does not alter the conclusions of the study. The HTML and PDF versions of this article have been corrected. The error remains only in PDFs downloaded before October 12, 2026.

**Figure:**
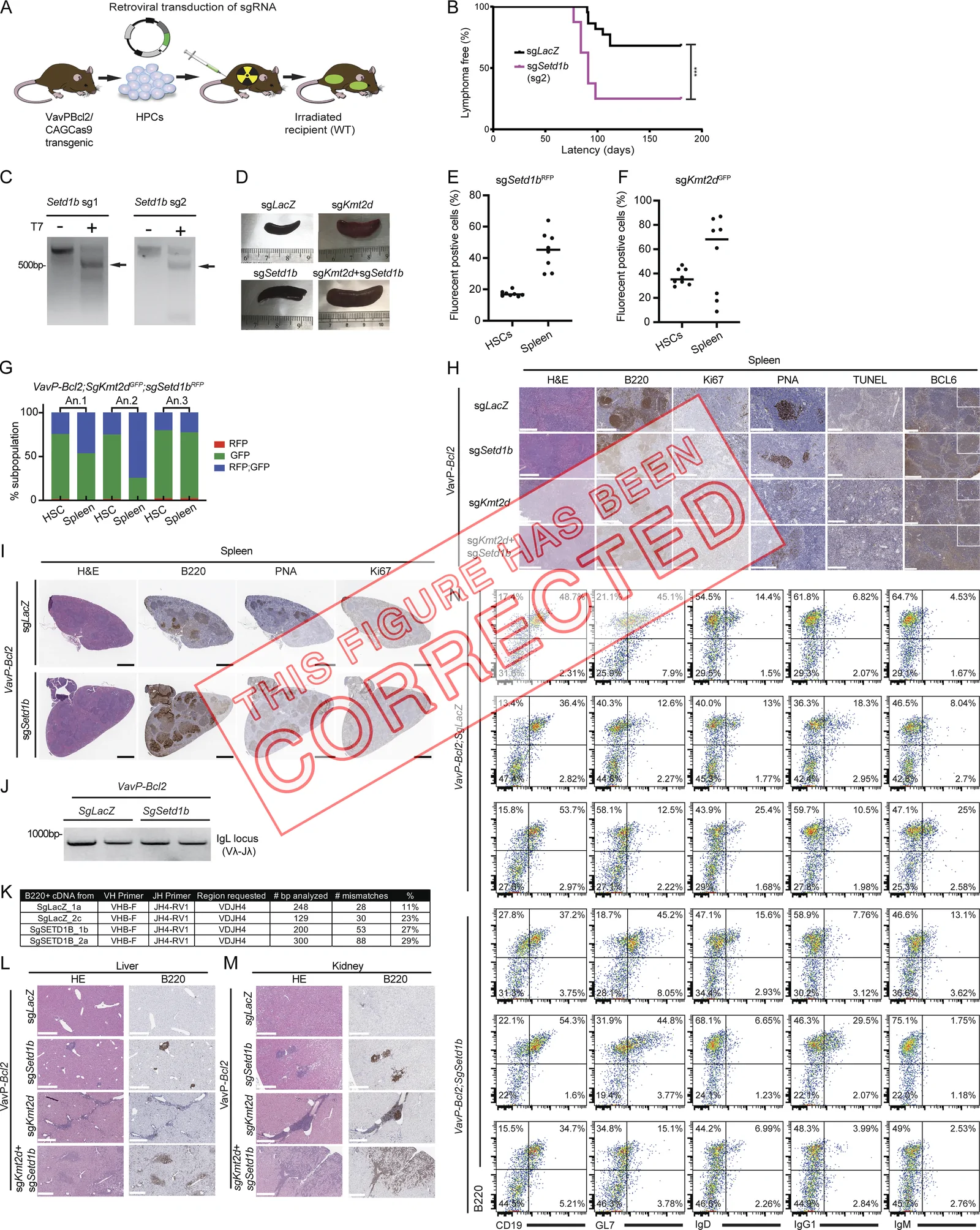


**Figure S3.**
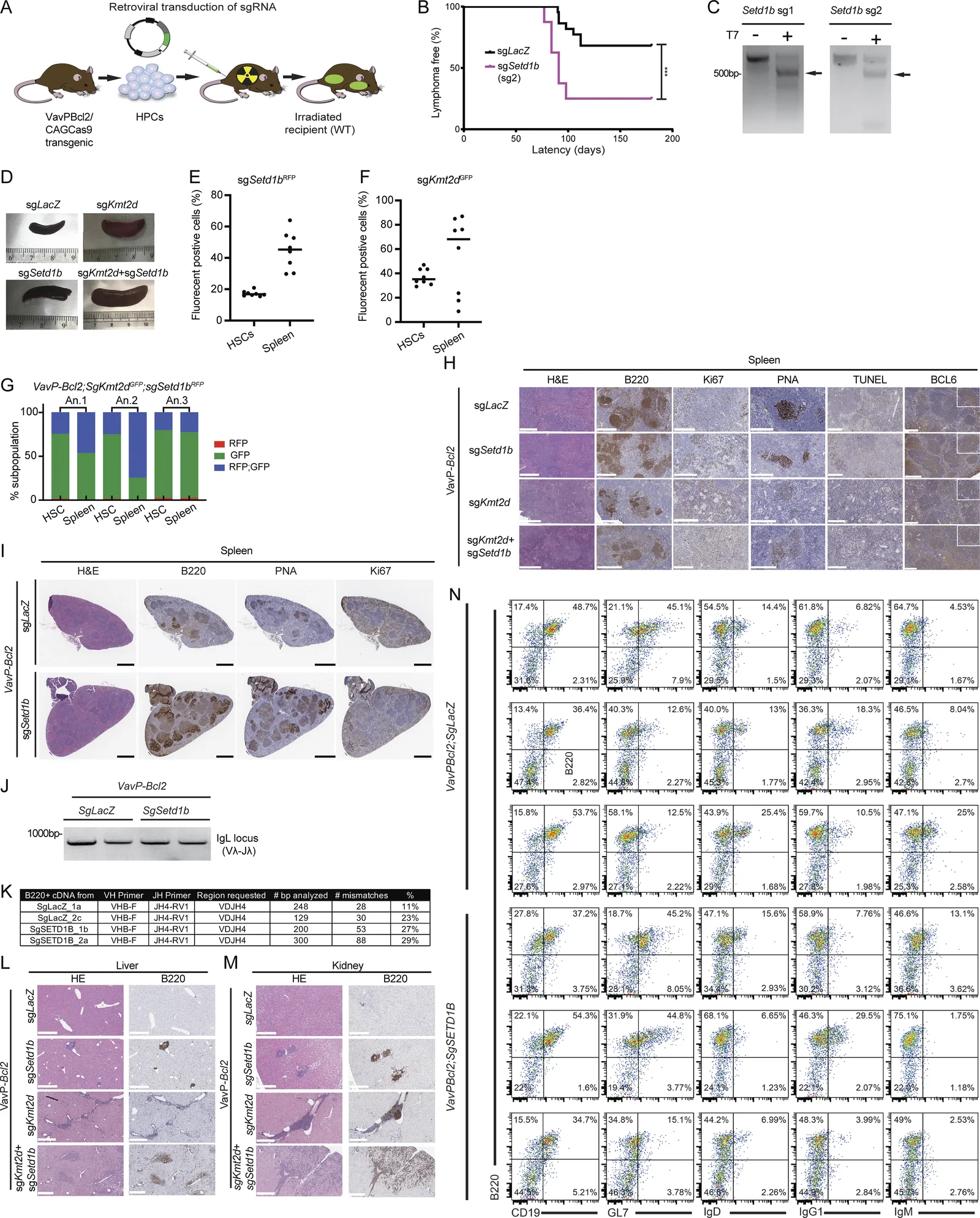
**Combined loss of *SETD1B* and *KMT2D* leads to a more aggressive disease. (A)** Illustration of FL adoptive transfer model based on the VavP-*BCL2* transgenic and retroviral transduction of HPCs followed by reconstitution in lethally irradiated, syngeneic, female mice. **(B)** Survival curves for mice harboring sgRNA targeting LacZ (*sgLacZ*) and mice carrying sgRNA targeting Setd1b (sg*Setd1b*) (***P = 0.005). P value was calculated using log-rank (Mantel–Cox) test. **(C)** Detection of genomic editing of Setd1b sgRNAs by T7 endonuclease I (T7E1) assay. The target fragments were amplified by PCR from genomic DNA from mouse tumors. Arrows indicate the digested fragments by T7EI. Data representative of at least two independent experiments are shown. **(D)** Representative spleens taken from each mouse group. Spleens from VavP-*Bcl2*; *sgSetd1b* (*n* = 27), VavP-*Bcl2*; *sgKmt2d* (*n* = 8), and VavP-*Bcl2*; *sgkmt2d+sgSetd1b* (*n* = 6) were enlarged compared with the control group, VavP-*Bcl2*; *sgLacZ* (*n* = 22). **(E and F)** Dot plot graph showing the percentage of positive transduced VavP-*Bcl2* HSCs (prior to injection into mice) and splenic lymphoma cells (after injection and disease), indicating cells harboring (E) sg*Setd1b-RFP* (sg*Setd1b*^*RFP*^) or (F) sg*Kmt2d-GFP* (sg*Kmt2d*^GFP^) plasmids, (*n* = 8). **(G)** Stacked bar graph showing RFP and GFP-positive transduced VavP-*Bcl2* HSCs (prior to injection into mice) and splenic lymphoma cells (after injection and disease), indicating cells harboring sg*Setd1b*^*RFP*^, sg*Kmt2d*^GFP^ plasmids or both sg*Setd1b*^*RFP*^ and sg*Kmt2d*^GFP^ (RFP; GFP) (*n* = 3). **(H)** Additional immunohistochemistry serial micrographs of VavP-*Bcl2* spleen tissues extracted from recipient mice upon sacrifice. VavP-*Bcl2*; *sgLacZ*, VavP-*Bcl2*; *sgSetd1b*, VavP-*Bcl2*; *sgKmt2d*, and VavP-*Bcl2*; *sgSetd1b+Kmt2d* were stained with H&E, B220, Ki67, PNA, TUNEL, and BCL6 of the liver tissues extracted from recipient mice upon sacrifice (*n* = 3 per group). Insets are 3× magnified. Scale bar: 200 μm, 500 μm. **(I)** Representative histological micrographs of VavP-*Bcl2*; sgLacZ versus VavP-*Bcl2*; sgSetd*1b*, stained with H&E, B220, PNA, and Ki67 (*n* = 3 per group). Scale bar: 1 mm. **(J)** DNA gel representing Ig heavy chain variable region (IgVH) rearrangements in B220^+^ cells isolated from VavP-*Bcl2*;sg*LacZ* and VavP-*Bcl2*;sg*Setd1b* spleens. Data representative of two independent experiments are shown. **(K)** Table summarizing the results of SHM analysis in DNA collected from VavP-*Bcl2*;sg*Setd1b* to VavP-*Bcl2*;sg*LacZ* tumors. **(L and M)** H&E and B220 staining of kidney (M) and liver (L) tissues extracted from recipient mice upon sacrifice. *n* = 3 per group. **(N)** Flow cytometry analysis of the cellular composition of splenic tumor cells from three mice in each genotype, comparing VavP-*Bcl2*;sg*Setd1b* to VavP-*Bcl2*;sg*LacZ*. Source data are available for this figure: SourceData FS3.

